# Examining the Relationships Between Job Characteristics, Emotional Regulation and University Teachers’ Well-Being: The Mediation of Emotional Regulation

**DOI:** 10.3389/fpsyg.2020.01727

**Published:** 2020-08-28

**Authors:** Jiying Han, Hongbiao Yin, Junju Wang

**Affiliations:** ^1^School of Foreign Languages and Literature, Shandong University, Jinan, China; ^2^Department of Curriculum & Instruction, The Chinese University of Hong Kong, Shatin, China

**Keywords:** emotional job demands, teaching support, emotional regulation strategies, well-being, ill-being, the job demands-resources model

## Abstract

This study investigated the associations between university teachers’ emotional job demands, teaching support, and well-being, and examined the mediating effect of emotional regulation strategies (i.e., reappraisal and suppression) in the job demands-resources (JD-R) model. The results of a survey of 643 university teachers in mainland China indicated that emotional job demands and teaching support, which facilitated teachers’ use of reappraisal strategies, had desirable effects on their well-being. Reappraisal was beneficial to teachers’ well-being, and suppression was harmful. These findings support the mediation role of emotional regulation, and evidence the applicability of the JD-R model to a higher education context.

## Introduction

The Job Demands-Resources (JD-R) model is a powerful framework explaining the relationships between job characteristics and employees’ performance and well-being (Bakker and Demerouti, 2007, 2014, 2017). The model classified job characteristics into two categories which are negatively correlated with each other: job demands and job resources. These two types of job characteristics are respectively assumed to have direct relationships with employees’ stress, motivation, health problems, and some organizational outcomes (Demerouti et al., 2001; Bakker et al., 2004). As previous studies have applied the JD-R model in a number of fields other than education, recent studies attempted to use this model in school (e.g., Hakanen et al., 2006; Simbula, 2010; Huang et al., 2019) and higher education settings (e.g., Boyd et al., 2011; Han et al., 2019).

Teaching is an emotional endeavor (Chang, 2009), and it is therefore important for teachers to regulate their emotions for effective classroom management and their well-being (Sutton et al., 2009; Yin, 2016). Although research into teachers’ emotional regulation and its effect on teachers’ well-being has received increasing attention in recent years (e.g., Yin, 2015; Wang et al., 2019; Yin et al., 2019), it remains an underexplored issue in the field of education. Based on the JD-R model, some studies have explored the role of emotional regulation in teachers’ work (Sutton, 2004; Brackett et al., 2010) and the relationships between teachers’ emotional regulation, job characteristics, and well-being among primary or secondary school teachers (Yin et al., 2016). However, there are few examinations of those relationships based on samples of university teachers.

Therefore, this present study aims at examining the relationships between two job characteristics of university teaching (i.e., emotional job demands and teaching support), university teachers’ emotional regulation strategies (i.e., reappraisal and suppression), and their well-being, with a particular focus on the mediating role of emotional regulation.

## Literature

### The JD-R Model and University Teachers’ Well-Being

The JD-R model is a well-known framework used to explore the relationships between job characteristics and employees’ well-being and performance. According to the JD-R model, there are two categories of job characteristics: job demands and job resources. Job demands are defined as job characteristics that “require sustained physical and/or psychological (cognitive or emotional) effort or skills and are therefore associated with certain physiological and/or psychological costs.” Job resources refer to job characteristics that are either “functional in achieving work goals,” or which can “reduce job demands and the associated physiological and psychological costs” (Bakker and Demerouti, 2007, p. 312).

Based on such a distinction, the JD-R model is conceptualized as a dual process model including two parallel processes. One is the health impairment process through which job demands exhaust employees’ mental and physical resources and hence lead to the depletion of individual energy (i.e., a state of exhaustion) and fatigue after-effects (Demerouti et al., 2001). Therefore, job demands are assumed to be a negative predictor of employees’ well-being and performance, and a number of empirical studies have proven the negative relationships between job demands and employees’ emotional exhaustion and burnout (Bakker and Demerouti, 2007; Schaufeli and Taris, 2014). The other is the motivational process through which the intrinsic and extrinsic motivational potentials of job resources lead to high work engagement, low cynicism, and excellent performance (Bakker and Demerouti, 2007). Specifically, job resources may act as an intrinsic motivation to promote individuals’ personal growth and learning, and as an extrinsic motivation to achieve work goals instrumentally (Demerouti et al., 2001). Job demands and resources are correlated with each other and may interact during the two development processes of job stress and job motivation (Bakker and Demerouti, 2014, 2017). Several studies have been conducted to test the application of the JD-R model among Chinese university teachers (Han et al., 2019, 2020). However, those studies have perceived teachers’ occupational stress as a main source of job demands, and very little is known about university teachers’ emotional job demands.

In this study, the emotional job demands of university teaching (EJD-UT) and teachers’ perceived teaching support were used as the indicators of job demand and job resource, respectively. Emotional job demands are qualitative demands imposed by interpersonal interactions of one’s job (Brotheridge and Lee, 2002). Emotional job demands are usually stressful and detrimental and therefore lead to unpleasant feelings (Grandey, 2000), because meeting those demands may lead to the depletion of resources and individual value. In educational settings, the emotional job demands of teaching derive from teachers’ interactions with students, colleagues, and administrators, and these emotional job demands refer to the specific requirements of teaching on teachers’ emotional expressions, e.g., suppressing negative emotions and showing positive emotions (Yin and Lee, 2012). Job resources, in the JD-R model, denote external resources including organizational resources, social resources, and task-related resources (Bakker and Demerouti, 2007). Scholars (Chang et al., 2010) have proposed conceptualizing teaching support in higher education at three levels: university teachers’ perceived teaching resources in the university, peer support from colleagues, and administrative support from the university. Teaching resources provide teachers with favorable working conditions, among which peer support from colleagues is a significant resource helping teachers achieve their work goals, and administrative support from the organization may help teachers deal with university teaching demands and illness (Väänänen et al., 2003; Bakker and Demerouti, 2007).

Teacher well-being was assessed by Warr’s two-axis model of workplace well-being (Warr, 1990): anxiety-contentment, and depression-enthusiasm. Both axes have a continuum of a psychological state anchored between pleasure and arousal (Yin et al., 2018). Anxiety and depression reflect an unpleasant and activated state. Contentment and enthusiasm reflect a pleasant and deactivated state. Considerable evidence of validity and reliability exists to support this approach for the assessment of well-being (de Jonge and Schaufeli, 1998; Mäkikangas et al., 2007; Huang et al., 2019).

### Emotional Regulation as a Mediating Process

The JD-R model is helpful for explaining the relationships between job characteristics and employees’ well-being. However, as most psychological approaches are based on the assumption that human behavior is a result of the interaction between environmental and personal factors, “personal resources” were recently integrated into the model (Schaufeli and Taris, 2014; Bakker and Demerouti, 2017). Personal resources denote the psychological characteristics or aspects of the self that are related to individuals’ ability to successfully control and affect their environment (Bakker and Demerouti, 2017). Review studies have indicated that personal resources mediate the relationships between job characteristics and well-being (Bakker and Demerouti, 2014, 2017). The mediation role of personal resources was recently supported by empirical studies conducted in mainland China. For example, as indicators of personal resources, teacher efficacy was a proven mediator among university teachers (Han et al., 2019), and emotional regulation was one among school teachers (Yin et al., 2016). These studies provide supporting evidence for a hypothesized mediation role of emotional regulation among university teachers.

In this present study, emotional regulation was used as a mediator in the relationships between university teachers’ job characteristics and well-being. Teachers’ emotional regulation indicates their ability to successfully influence their emotions in the workplace and interact with their work environment. Teachers’ emotional regulation strategies, resulting from the interaction between environmental and personal factors, may further influence their well-being. Gross proposed two broad types of emotional regulation strategies: cognitive reappraisal and expressive suppression. The former is an antecedent-focused emotional regulation that involves “construing a potentially emotional-eliciting situation in non-emotional terms,” and the latter is a response-focused emotional regulation that involves “inhibiting ongoing emotional expressive behavior” (Gross, 2002, p. 283). This distinction is consistent with Lazarus and Folkman’s (1984) distinction between problem-focused and emotional-focused coping strategies in the face of the emotional demands of work. Based on the cognitive-phenomenological theory of coping, Lazarus and Folkman (1984) proposed two major coping strategies: coping that changed the cause of the stress, and that managed the subsequent emotion. So far there have been consistent findings that the former are active coping strategies producing more favorable outcomes and the latter are negative or avoidant coping strategies leading to increased depression and anxiety (Kim et al., 2010).

In line with the distinction between resources and demands (Bakker and Demerouti, 2017), Bakker and Demerouti (2017) suggested that the JD-R model could be further expanded to include personal demands, which might be involved in both the health-impairment process and the motivational process proposed by the JD-R model. Personal demands are defined as “the requirements that individuals set for their own performance and behavior that force them to invest effort in their work and are therefore associated with physical and psychological costs” (Barbier et al., 2013, p. 751). According to Yin et al. (2018), reappraisal is considered as a personal resource, which reflects individuals’ ability to efficiently control their emotions and adapt themselves to an environment. Suppression is viewed as a personal demand denoting individuals’ inability to cope with an emotionally demanding environment, and it requires extra effort and physical or psychological costs. Recent empirical studies have consistently revealed the beneficial effects of reappraisal and the detrimental effects of suppression on well-being indicators in school settings (e.g., Jiang et al., 2016; Yin et al., 2016, 2018).

### Teacher Emotion in the Context of Chinese Higher Education

As well as schools, universities are complex emotional arenas where teachers tend to be exposed to the emotional demands of teaching. Studies of teacher emotion in higher education had not received adequate research attention until the 2010s. A few studies were conducted in several cultural contexts including Australia (Trigwell, 2012), the United Kingdom (Bennett, 2014), and China (Zhang and Zhu, 2008; Zhang et al., 2019). These limited studies provided preliminary evidence for understanding the emotional process in relation to university teachers’ well-being (i.e., burnout and satisfaction) and teaching behaviors (e.g., teaching styles and approaches to teaching). However, the literature on teacher emotions in higher education would be greatly enriched by studies involving more important psychological constructs (Zhang et al., 2019).

The perception of emotion varies across cultures (Krone and Morgan, 2000), and Chinese cultural values play a crucial role in shaping and regulating peoples’ emotions (Zhang and Zhu, 2008). For example, the traditional Chinese value of collectivism and the interdependent view of self emphasize the maintenance of harmonious relationships, Chinese people tend to neutralize their inner feelings to avoid negative emotions and to save face (Krone et al., 1997). Meanwhile, as the traditional Chinese conceptions of teaching endow Chinese teachers with the dual roles of authorities of knowledge and models of behavior, the interaction between Chinese university teachers and students is characterized by teachers’ humanistic concern for students and close teacher-student relationships (Han et al., 2016).

In addition, the rapid expansion of higher education in China since 1999, the shift toward a greater emphasis on research, and the diversification of the motivation of students have brought heavy workload and pressure for university teachers (Shen and Xiong, 2015). As a result, university teachers have had increased job demands imposed on them, creating a considerable amount of pressure. These demands may serve as external stimuli, triggering teachers’ appraisal of their situation as stressful and the adoption of subsequent coping strategies. Accordingly, teachers may use either reappraisal or suppression strategies to cope with the emotional demands. Meanwhile, when university teachers are provided with teaching support at different levels, they may feel less stressed and have less need to inhabit the ongoing emotion. Different emotional regulation strategies produce different consequences. This study aims to integrate emotional regulation strategies, as mediating processes, into the JD-R model. With a sample of Chinese university teachers, the study examined the relationships between university teachers’ perceived emotional job demands and teaching support, emotional regulation strategies (reappraisal and suppression), and their well-being.

Based on the reviewed literature on the JD-R model, emotional regulation theory, and the evidence from empirical research, the following hypotheses were established.

H1. The emotional job demands of university teaching are positively related to teacher ill-being (H1a) and negatively related to teacher well-being (H1b);
H2. The perceived teaching support is negatively related to teacher ill-being (H2a) and positively related to teacher well-being (H2b);
H3. The emotional job demands of university teaching are positively related to reappraisal (H3a) and suppression (H3b);
H4. The perceived teaching support is positively related to reappraisal (H4a) and negatively related to suppression (H4b);
H5. Reappraisal is negatively related to teacher ill-being (H5a) and positively related to teacher well-being (H5b);
H6. Suppression is positively related to teacher ill-being (H6a) and negatively related to teacher well-being (H6b);
H7. Reappraisal mediates the effect of emotional job demands of university teaching on teacher ill-being (H7a) and well-being (H7b);
H8. Suppression mediates the effect of emotional job demands of university teaching on teacher ill-being (H8a) and well-being (H8b).

**FIGURE 1:**
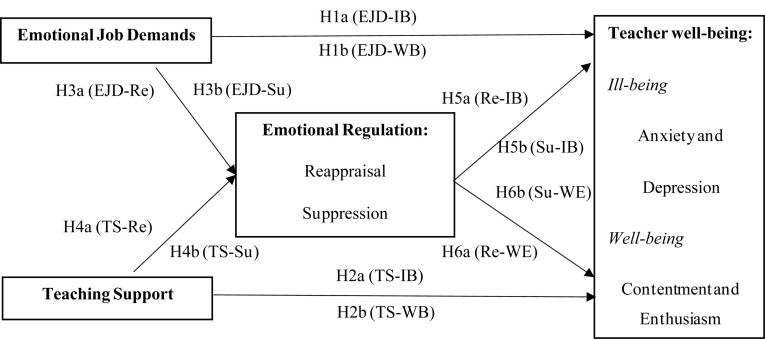
The hypothesized model. EJD, emotional job demands; TS, teaching support; Re, Reappraisal; Su, Suppression; IB, ill-being; WB, well-being.

Figure 1 presents the hypothesized model to be tested in this study.

## Materials and Methods

### Participants

Following institutional review board procedures, this study was conducted according to the recommendations of the Survey and Behavioral Research Ethics Committee at the Chinese University of Hong Kong with written informed consent from all participants.

The survey was conducted in December 2018. Convenience sampling was used to collect data during a university teacher training program which was initiated by Shandong Provincial Education Department. A total of 1,000 copies of the questionnaire were distributed to teachers from the public higher education institutions (HEIs) of Shandong, a developed province in East China. All teachers were invited to voluntarily participate in the paper-based questionnaire. With a response rate of 65.4%, the sample of this study consisted of 643 university teachers from 50 HEIs ranging from national key research-oriented universities to local vocational institutions of higher education. 62.9% of the sample were male. Approximately 7.9% were teaching assistants (the beginning professional rank of HEIs in China), 23.2% were lecturers, 58.9% were associate professors, and 10% were professors. Regarding disciplinary background, 39.3% were from the liberal arts, 14.2% were from science, 40% were from technology, and 39% were from medical science.

### Measures

The questionnaire consisted of 39 items pertaining to four measures, and it required approximately eight minutes to complete on average. As the original measures were scored differently and consistently shown to be valid and reliable in previous studies, we respectively preserved their original scale formats to elicit reliable responses.

#### Emotional Job Demands of University Teaching Scale

The EJD-UT were measured by a 6-item unidimensional scale which was adapted from Yin’s (2015) Emotional Job Demands of Teaching Scale (EJD-T). Item 1 (“I perform my teaching well, I have to spend most of my time interacting with others (e.g., students and colleagues”) and item 4 (“I have to use my emotions and behaviors to create a reassuring climate for my students”) of the original EJD-T were rephrased to be more suitable to the university teaching context by eliminating the concern of parents. Meanwhile, two additional items were added. They were “In university teaching, I have to stimulate and elicit students’ emotions so that they can devote themselves to learning” and “In university teaching, I have to manage my emotions and create an atmosphere facilitating students’ learning.” All items were scored using the original 5-point Likert scale format ranging from 1 (strongly disagree) to 5 (strongly agree).

#### The Revised Faculty-Perceived Teaching Support Scale

The nine-item Revised Faculty-Perceived Teaching Support scale (R-FPTS) adapted by Han et al. (2018) was used to assess teachers’ perceived teaching support. This is a shortened scale of the original 14-item FPTS developed by Chang et al. (2010), and the adaptation of the scale was based on results of a series of exploratory factor analyses when it was firstly used in a Chinese context (Han et al., 2018). The scale consists of three subscales, each of which comprises three items: teaching resources (e.g., “The university provides the facilities and resources for teaching”), administrative support (e.g., “The administrators care about teachers’ teaching effectiveness”), and peer support (e.g., “The colleagues provide teaching demonstration opportunities for me to observe other colleagues’ teaching”). The items were scored on a 4-point scale ranging from 1 to 4, with higher scores indicating university teachers’ higher perceptions of teaching support.

#### Emotional Regulation Questionnaire

The Emotional Regulation Questionnaire (ERQ) developed by Gross and John (2003) was used to assess university teachers’ emotional regulation strategies. The ERQ is a 10-item scale measuring reappraisal strategies (six items, e.g., “When I’m faced with a stressful situation, I make myself think about it in a way that helps me stay calm”) and suppression strategies (four items, e.g., “When I am feeling positive emotions, I am careful not to express them”). We have added “in university teaching” to each item to make sure that the participants would respond to the items according to their teaching experiences. All items were scored on a 5-point Likert scale ranging from 1 (strongly disagree) to 5 (strongly agree).

#### Occupational Well-Being Scale

Warr’s (1990) 12-item Occupational Well-Being Scale (OWS) was used to assess university teachers’ well-being. The scale was designed to assess teachers’ well-being in two dimensions: ill-being and well-being. The participants were required to respond to their job-related anxiety (tense, uneasy, and worried), contentment (calm, contented, and relaxed), depression (depressed, gloomy, and miserable) and enthusiasm (cheerful, enthusiastic, and optimistic) in the past few weeks. The responses ranged from 1 (never) to 6 (all of the time).

### Data Analysis

SPSS 22.0 and Mplus 7.0 were used to analyze the data. The descriptive statistics (mean and standard deviation) and correlations were calculated using SPSS. Confirmatory factor analysis (CFA) and structural equation modeling (SEM) were conducted using Mplus to test the hypotheses. Mediation analysis based on 5000 bootstrapping samples was used to examine the mediation role of reappraisal and suppression in the hypothesized model. The acceptance of models was based on the following goodness-of-fit statistics: a Comparative Fit Index (CFI) and Tucker–Lewis Index (TLI) of no less than.90, and a root mean square error of approximation (RMSEA) of no more than.08 (Schreiber et al., 2006).

## Results

### Construct Validity, Reliability, Descriptive Statistics, and Correlations

Confirmatory factor analysis was used to test the factor structure of each measure. The measures of both teaching support (χ^2^ = 84.99, df = 24, *p* < 0.01, CFI = 0.98, TLI = 0.98, RMSEA = 0.063) and occupational well-being (χ^2^ = 203.33, df = 48, *p* < 0.01, CFI = 0.97, TLI = 0.96, RMSEA = 0.071) exhibited good fit with the factor structures from prior research. Factor loadings of teaching support ranged from 0.53 to 0.94, and those of occupational well-being ranged from 0.66 to 092. However, the original factor solutions revealed very high inter-correlations between contentment and enthusiasm (*r* = 0.91, *p* < 0.001) and between anxiety and depression (*r* = 0.72, *p* < 0.001), indicating potential overlaps of these scales. To address this problem, we further constructed a higher-order model with two factors, well-being (contentment and enthusiasm) and ill-being (anxiety and depression). The higher-order two-factor solution exhibited an acceptable model fit (χ^2^ = 248.46, df = 49, *p* < 0.01, CFI = 0.97, TLI = 0.96, RMSEA = 0.073).

The original factor solutions for both emotional job demands and emotional regulation raised questions about lower factor loading and common shared meaning. We re-conducted the CFA models. The acceptable model fit of emotional job demands was obtained by deleting item 1 (“To teach well, I have to be considerate and think from the view of point of my students and colleagues”) which was below 0.4. The second CFA was conducted after deleting item 1 indicated a good model fit (χ^2^ = 15.55, df = 4, *p* < 0.01, CFI = 0.99, TLI = 0.97, RMSEA = 0.067). Factor loadings of the remaining five items ranged from.47 to.82. Similarly, CFA results of emotional regulation revealed a good model fit (χ^2^ = 61.62, df = 19, *p* < 0.01, CFI = 0.93, TLI = 0.90, RMSEA = 0.059) after we deleted item 1 (“When I want to feel a more positive emotion, such as joy or amusement, I change what I’m thinking about”) for its lower factor loading and item 9 (“When I am feeling negative emotions, I make sure not to express them”) for its shared meaning with item 2. Factor loadings of the remaining eight items ranged from 0.41 to 0.80.

Table 1 shows the descriptive statistics of all factors, reliability, and correlation coefficients between the latent factors. The internal consistency of all measures was within acceptable limits, ranging from 0.53 to 0.90. Four factors were positively associated with reappraisal (emotional job demands, *r* = 0.15, *p* < 0.01; teaching support, *r* = 0.12, *p* < 0.01; suppression, *r* = 0.24, *p* < 0.01; well-being, *r* = 0.19, *p* < 0.01). Teaching support was positively related to well-being (*r* = 0.40) and negatively related to ill-being (*r* = −0.24, *p* < 0.01). Suppression was positively associated with suppression (*r* = 0.14, *p* < 0.01) and well-being (*r* = −0.45, *p* < 0.01).

**TABLE 1:** Descriptive statistics, Cronbach’s α and correlation matrix of the variables.

	1	2	3	4	5	6
1 Emotional job demand	(0.77)					
2. Teaching support	0.07	(0.90)				
3. Reappraisal	0.15**	0.12**	(0.65)			
4. Suppression	−0.03	−0.01	0.24**	(0.53)		
5. Ill-being	0.03	−0.24**	−0.03	0.14**	(0.90)	
6. Well-being	0.07	0.40**	0.19**	−0.01	−0.45**	(0.90)
*M*	4.16	4.13	3.69	2.98	2.23	3.33
SD	0.47	0.88	0.44	0.61	0.80	0.74

**p < 0.01; Cronbach’s α reliability coefficients in parentheses along the diagonal.

### SEM Analysis

**FIGURE 2:**
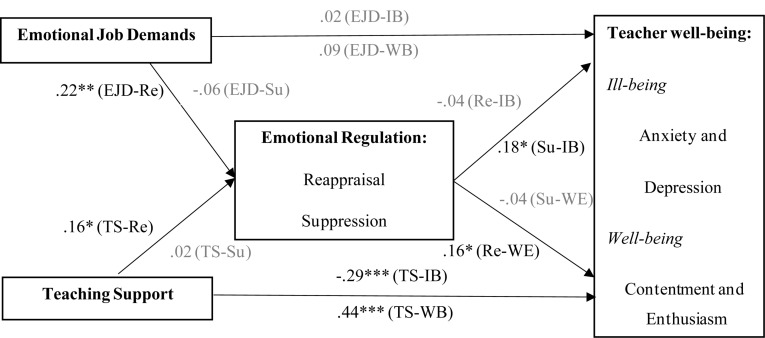
SEM results of the hypothesized model (*N* = 643). Goodness-of-fit indices: χ^2^ = 1249.59, df = 506, *p* < 0.01, CFI = 0.93, TLI = 0.93, RMSEA = 0.053.

Structural equation modeling was constructed to test the relationships between emotional job demands, teaching support, emotional regulation, and occupational well-being. Emotional job demands and teaching support were primary independent variables, and well-being and ill-being were dependent variables. Reappraisal and suppression were mediators between the independent and dependent variables. The full model exhibited an acceptable model fit with the data (χ^2^ = 1249.59, df = 506, *p* < 0.01, CFI = 0.93, TLI = 0.93, RMSEA = 0.053), and the path diagram between these constructs along with their respective path coefficients are presented in Figure 2.

As Figure 2 illustrates, teaching support was negatively related to teacher ill-being (*β* = −0.29, *p* < 0.001) and positively related to teacher well-being (*β* = 0.44, *p* < 0.001), supporting H2a and H2b. Both EJD-UT (*β* = 0.22, *p* < 0.01) and teaching support (*β* = 0.16, *p* < 0.05) were positively related to reappraisal, supporting H3a and H4a. Reappraisal was positively related to teacher well-being (*β* = 0.16, *p* < 0.05), and suppression was positively related to teacher ill-being (*β* = 0.18, *p* < 0.05), supporting H5b and H6a.

### Mediation Analysis

To examine the mediation role of reappraisal and suppression in the hypothesized model, mediation analysis based on 5000 bootstrapping samples was conducted. As Hayes (2009) indicated, an indirect effect is significant if zero is not located between the lower level and the upper level of the CI. The results of the mediation analysis (see Table 2) indicated that, of the hypothesized mediation effects, reappraisal mediated the effect of EJD-UT on teacher well-being, supporting H7b. However, contrary to our expectations, no convincing empirical support was found in the mediation effect of suppression on the relationships between university job characteristics and teacher well-being. For purposes of brevity, the non-significant findings concerning suppression are not reported. Table 3 summarizes the results of the hypothesis tests.

**TABLE 2:** The estimates of direct effects and indirect effects of the 95% confidence intervals.

Dependent Variable	Independent Variable	Mediator	Direct Effect	Indirect Effect	95% CIs		R^2^
					Lower 2.5%	Upper 2.5%	
Well-being	Emotional job demands		0.09	0.04	0.00	0.08	0.26
		**Reappraisal**		0.04	0.01	0.07	
		Suppression		0.00	-0.01	0.02	

*Bootstrap samples = 5000. Bold items showing significant mediation effect.*

**TABLE 3:** Summary of hypothesis tests.

No.	Hypothesis	Results
H1a	Emotional job demands are positively related to teacher ill-being.	Not supported
H1b	Emotional job demands are negatively related to teacher well-being.	Not supported
**H2a**	**The perceived teaching support is negatively related to teacher ill-being.**	**Supported**
**H2b**	**The perceived teaching support is positively related to teacher well-being.**	**Supported**
**H3a**	**The emotional job demands of university teaching are positively related to reappraisal.**	**Supported**
H3b	The emotional job demands of university teaching are positively related to suppression.	Not supported
**H4a**	**The perceived teaching support is positively related to reappraisal.**	**Supported**
H4b	The perceived teaching support is negatively related to suppression.	Not supported
H5a	Reappraisal is negatively related to teacher ill-being.	Not supported
**H5b**	**Reappraisal is positively related to teacher well-being.**	**Supported**
**H6a**	**Suppression is positively related to teacher ill-being.**	**Supported**
H6b	Suppression is negatively related to teacher well-being.	Not supported
H7a	Reappraisal mediates the effect of emotional job demands on teacher ill-being.	Not supported
**H7b**	**Reappraisal mediates the effect of emotional job demands on teacher well-being.**	**Supported**
H8a	Suppression mediates the effect of emotional job demands on teacher ill-being.	Not supported
H8b	Suppression mediates the effect of emotional job demands on teacher well-being.	Not supported

*Bold items showing supported hypotheses.*

## Discussion

This study sought to contribute empirical and theoretical knowledge to the applicability of the JD-R model in the context of higher education, especially by integrating emotional regulation strategies into the JD-R model as mediator. The study is an important step toward understanding mechanisms of university teacher well-being with an intervention of effective emotional regulation strategies. Nearly half of our expected pathways were supported in the full SEM, and reappraisal was found to play a significant mediation role in the relationship between EJD-UT and teacher well-being.

### Theoretical Implications

Firstly, the study provides evidence of the applicability of the JD-R model in the context of higher education. The JD-R model postulates that both job demands and job resources are significant predictors of employees’ organizational performance. Our results indicate that both emotional job demands and teaching support had desirable effects on university teachers’ well-being. However, the former exerted its effect in an indirect way, while the latter had both direct and indirect beneficial effects. On one hand, empirical studies have reached a consensus that emotional job demands exhibit an important direct effect on unpleasant job outcomes such as emotional exhaustion, burnout, job dissatisfaction, and ill-being (e.g., Richardson et al., 2008; Yin et al., 2019). On the other, previous study has revealed an indirect effect of university teachers’ job demands on positive outcomes, i.e., work engagement, through the mediation of personal resources (Han et al., 2019). This is consistent with the present study indicating that emotional job demands exerted an indirect positive effect on teacher well-being (contentment and enthusiasm) through the mediation of reappraisal. As suggested, this is probably because when teachers perceive that their emotional job demands could be handled with appropriate coping strategies, they feel more confident in their efforts to meet those demands, leading to positive outcomes. This highlights the significant role of reappraisal, as a personal resource, in boosting the desirable impact of emotional job demands on well-being.

Although both reappraisal and suppression are strategies for coping with emotionally stressful conditions (Lazarus, 1993), our results provide empirical evidence for reappraisal, rather than suppression, as a more adaptive and effective strategy for university teachers to manage emotions in the classroom. According to Joseph and Newman (2010), those who are competent at regulating emotion often engage themselves in a more effective strategy, that is, cognitive reappraisal. The use of suppression tends to reveal a lack of internal regulatory ability and requires extra effort from teachers (Bakker and Demerouti, 2017). As emotional job demands in higher education are changing, being emotionally competent is part of the professional skills for university teachers (Lawless and Brandi, 2018). Therefore, unlike school teachers who have reported using prominent maladaptive strategies of suppression (Yin et al., 2018), we may conclude that university teachers are more likely to regulate their emotions by adopting adaptive and effective strategies to cope with their perceived emotional job demands.

Secondly, our results revealed the positive effect of emotional job demands on reappraisal and a lack of significant effect on suppression. Although both reappraisal and suppression are coping strategies sensitive to emotional job demands, they are different in nature. In line with the distinction between personal resource and personal demand, a further examination indicated that reappraisal was associated with better interpersonal functioning and abilities to deal with stress-provoking situations, so reappraisal could efficiently change the entire subsequent emotion trajectory by successfully reducing the negative emotion. In contrast, suppression may not be helpful in reducing the negative emotion because it requires the individual’s extra effort to manage emotion response tendencies. These repeated efforts may consume cognitive resources and lead to negative feelings (Gross and John, 2003; Yin et al., 2018). Therefore, the significant effect of emotional job demands on reappraisal rather than suppression indicates that it is less likely for university teachers to suppress their feelings during teaching because they may have more autonomy in emotional activities compared with school teachers.

### Practical Implications

Existing studies framed by the JD-R model are relatively scarce in China compared to the intensive research in Western countries. The results of this study not only supports the application of the JD-R model to the higher education context especially, particularly in a non-Western society, but also reveal some ways to improve university teachers’ well-being in practice.

Firstly, the findings of this study reveal the prominent role of job characteristics in enhancing university teachers’ well-being via a motivation process in the JD-R model, indicating that university teachers may be more competent at adopting effective coping strategies to deal with emotional stress. As emotion is still one of the most neglected issues in higher education research, our findings highlight the significance and need for faculty development programs to focus on teachers’ emotional demands from the perspective of teacher emotional development, despite the traditional preference for teachers’ cognitive and behavioral development. With the acknowledgment of the beneficial effect of emotional job demands of university teachers, a comprehensive conceptualization of faculty development is expected, and university teachers are expected to be aware of the emotional demands of university teaching, so that they could adopt more effective coping strategies in the stressful context.

Secondly, our study supported the inclusion of reappraisal as a personal resource in mediating the positive effect of job characteristics on teachers’ well-being. Although emotional job demands are commonly considered as a predictor of unpleasant job outcomes, this finding helps to identify the importance of equipping university teachers with specific antecedent-focused strategies for managing their emotions and reappraising the emotional stimulus in stress-provoking environments. University interventions are expected to provide individual teachers with healthier patterns of emotional regulation strategies, and to prepare teachers well for potentially emotional strains which may serve as an effective intervention before teachers’ emotion responses have been fully generalized. Scholars (e.g., Sutton and Harper, 2009; Yin, 2016) have identified different stages and specific strategies to regulate emotions, and these findings may serve as a foundation for planning effective interventions for university teachers to improve their well-being.

Thirdly, along with the assumptions of the JD-R model, this study suggests that creating a supportive and co-operative environment for university teaching is effective in terms of improving university teachers’ well-being. Regulations and measures aiming at improving teachers’ well-being may consider stimulating co-operation among teachers and the provision of sufficient teaching resources, such as facilities, technologies, and software resources.

## Limitations and Future Directions

This study offers insights into the applicability of the JD-R model in a Chinese context in higher education and the mediation role of emotional regulation in the relationship between job characteristics and teachers’ well-being. However, some limitations should be noted as indications for future research. A major limitation relates to the design and method of the present study because a cross-sectional design is insufficient to confirm the causal relationships between the constructs. Hence a longitudinal design may be considered in the future to clarify the directionality of the regression paths. Secondly, the results of this study are derived from the participants’ self-reports which might inflate the relationships between variables because of the shared method variance. Future studies may consider using a mixed-method design to triangulate data collected from multiple sources, and qualitative design is expected to drive further interpretations to inform internal complexities and interactions of the context. Thirdly, as the aim of this study is to examine the relationships between the variables, we did not address potential differences in teachers’ perceptions among those with different background information. Future research may advance this study and provide more descriptive data.

## Data Availability Statement

The datasets generated for this study are available on request to the corresponding author.

## Ethics Statement

The studies involving human participants were reviewed and approved by the Survey and Behavioral Research Ethics Committee. The patients/participants provided their written informed consent to participate in this study.

## Author Contributions

JH collected and analyzed the data and wrote the first draft of the manuscript. HY designed the research and wrote the first draft of the manuscript. JW helped with data collection and finalized the manuscript. All authors contributed to the article and approved the submitted version.

## Conflict of Interest

The authors declare that the research was conducted in the absence of any commercial or financial relationships that could be construed as a potential conflict of interest.
